# Distribution of metastasis in the brain in relation to the hippocampus: a retrospective single-center analysis of 565 metastases in 116 patients

**DOI:** 10.1186/s40644-019-0188-6

**Published:** 2019-01-22

**Authors:** Qian Sun, Min Li, Gengming Wang, Hongbo Xu, Zelai He, Yongchun Zhou, Yan Zhou, Yufu Zhou, Hongwei Song, Hao Jiang

**Affiliations:** 1 Department of Radiation Oncology, The First Affiliated Hospital of Bengbu Medical College, Bengbu, 233004 China; 2 Department of Pharmacy, Bengbu Medical College, Bengbu, 233004 China

**Keywords:** Hippocampal avoidance, Brain metastases, Distribution, Neurocognitive function

## Abstract

**Objective:**

To examine the distribution of brain metastases (BM) in relation to the hippocampus, so as to determine the risk of metastasis in the hippocampus, and thus provide experimental evidence for the hippocampal avoidance (HA) in patients with BM during radiotherapy.

**Methods:**

(1) For the retrospective analysis of 116 patients diagnosed with malignancies, confirmed as BM, from December 2014 to December 2016 at the First Affiliated Hospital of Bengbu Medical College. We obtained the T1-weighted, postcontrast axial, sagittal, and coronal Magnetic Resonance imaging (MRI) images f the patients, in supine position, using the head restraints and head thermoplastic masks to adjust the positioning, with computed tomography (CT) positioning scan ranging from the head to the mandible (layer thickness: 3 mm). CT and MRI images were fused on a Philips Pinnacle v9.8 treatment planning system;(2) Every metastasis of the 565 metastases was contoured;(3) hippocampus were contoured, and hippocampus with 5 mm expansion envelopes were analyzed;(4) Using the SPSS 16.0 software, we analyzed the relation between the distribution and age, sex, Karnofsky performance status (KPS), primary site, aggregate volume of intracranial metastases and the whole brain. The data were analyzed using a binary logistic regression analysis method, with two-sided *P* < 0.05 for statistical significance.

**Results:**

In this study, we recruited 116 patients with 565 metastases. Among them, 1.7% (*n* = 2) had metastases in the hippocampus, and 11.2% (*n* = 13) had metastases within 5 mm of the hippocampus, of which more than half were patients with non-small cell lung cancer (*n* = 7). Using a binary logistic regression to analyze the relation between the metastases located within 5 mm of the hippocampus and age (*P* = 0.395), sex (*P* = 0.139), KPS (*P* = 0.724), primary site (*P* = 0.894), aggregate volume of intracranial metastases (*p* = 0.093) and the whole brain (*p* = 0.998), and none of them showed statistically significant difference between them and the metastases location (P>0.05).

**Conclusion:**

This study showed a low risk for the perihippocampal metastases (PHM) and no significant correlation between PHM and age, sex, KPS, primary site, aggregate volume of intracranial metastases and the whole brain. Accordingly, it is may be acceptable to avoid the perihippocampal region during whole brain radiotherapy.

## Introduction

Whole-brain radiotherapy (WBRT) has been commonly applied in the clinic since the 1960s, including in the control of intracranial metastatic tumors and whole-brain preventive exposure. With the younger brain tumor patients and the extension of the lifetime of tumor patients, increasingly patients call for better treatment and high quality of life, thus people pay more attention to the neurocognitive function damage produced while using radiotherapy technology to treat tumors [1–3]. The RTOG 0933trial conducted by the Radiation Therapy Oncology Group (RTOG) of the American College of Radiology (ACR) included 113 patients with encephalic metastasis treated by WBRT, using 30Gy given over 10 daily fractions. The hippocampus and the 5-mm area around it were selected as the organs at risk. In the RTOG 0933 trial, the mean dose endangering organs was stipulated as< 10 Gy and the maximum dose as< 17 Gy. The results revealed that the occurrence of hypomnesis in patients after 4 months of radiotherapy was 7%, which was significantly lower than in the historical control group (30%) [4], showing that function of the hippocampus could be protected. How to alleviate neurocognitive function damage, while maximizing the beneficial effect of radiotherapy has become the current hot research topic in the medical profession.

As an important neural gyrus area in the brain temporal lobe, the hippocampal gyrus manages the primary memory that humans create or have managed in a short time. Once the hippocampal gyrus is damaged, patients are likely to suffer severe neurocognitive deficits, resulting in hypomnesis or even disorders involving impairment of the patients’ memory cognitive ability. Thus, protection and limitation of the clinical exposure dose of the hippocampal gyrus are taken seriously by exposure tumor experts. Scoville et al [5] reported memory impairment in patients whose bilateral temporal lobe was excised in the operation and emphasized that the reason for the severe and enduring hypomnesis was the wide excision of the hippocampus. Additionally, they speculated that maintenance of normal memory function should be connected with the normal hippocampal formation and function. The research of Gondi et al [6]indicated that after mammals received radiotherapy, neural dendritic cells under the lower dentate gyrus were involved in the remarkable dose-response reduction, including the dendritic arborization, quantity and volume. The hippocampus is closely related to the neurocognitive function of humans. The entirety of hippocampal formation plays a cruciall role in the formation of learning memory [7, 8]. Gondi et al. [6] used the Hopkins vocabulary learning test revision scale to evaluate 42 eligible patients for cognitive function of standardization and life quality, and found that: (1) the relevant hypomnesis in patients with whole brain exposure avoiding the hippocampus was significantly less than the whole brain exposure group; (2) Patients with whole brain exposure avoiding the hippocampus had 6.8 months of median survival time, which exceeded the historical control group (4.9 months) and the gamma knife combined with whole brain exposure group (5.7 months). The study by Chang [9] found that patients treated with SRS plus WBRT were at a greater risk of a significant decline in learning and memory function by 4 months compared with the group that received SRS alone. The study by Brown [10] found that among patients with 1 to 3 brain metastases, the use of SRS alone, compared with SRS combined with WBRT, resulted in less cognitive deterioration at 3 months. Therefore, it can be discerned that WBRT avoiding the hippocampus can reduce the damage to the hippocampal function and adjacent organs, while ensuring therapeutic dose.

A review of the relevant literature shows that 10–20% of malignant tumor patients will eventually develop intracranial metastatic tumors [11, 12]. However, most patients with intracranial metastatic tumors have poor prognosis and about 5% of patients ultimately die of metastatic encephaloma, instead of extracranial neoplasms [13]. At present, nuclear magnetic resonance enhanced scanning is considered the golden standard for the diagnosis of intracranial tumors.

Whole brain exposure is an effective therapeutic method to control intracranial metastatic tumors. Prophylactic cranial irradiation (PCI) that is applied in small cell lung cancer (SCLC) patients with complete remission of primary lesion can prolong the overall survival [14], but whole brain exposure can cause a series of toxic reactions, including degeneration of cognitive function and reduction of life quality [15]. Some studies showed that the probability of intracranial metastasis in the hippocampus is extremely lower than that in other parts [16, 17]. The study by Harth [16] found that the number of metastatic lesions in the hippocampus accounted for 0.4% of the total metastatic lesions. Relative to conventional WBRT, the radiotherapy avoiding the hippocampus will increase the recurrence risks by 0.4%. Also, there is almost no difference in the therapeutic outcome in non-small cell lung cancer (NSCLC) patients between these two radiotherapy technologies. Patients with oligonucleotide metastatic lesions (1–3 metastatic lesions) who receive WBRT avoiding the hippocampus have the fewer metastatic risks of damage to the hippocampus than patients with non-oligonucleotide metastatic lesions [18]. The research of Ghia et al. [19] indicated that a 5-mm margin around the hippocampus for conformal avoidance whole brain radiotherapy represents an acceptable risk. The studies showed that intensity modulated exposure therapy and helical tomotherapy can ensure that the hippocampus D40% < 7.3Gy. If the threshold is exceeded, hypomnesis in forward character memory will be caused [2]. Following the increased application of the hippocampal protective whole brain exposure technology in the clinic, the discussion of the relation between intracranial metastasis and hippocampus was further studied. In this research, the positional relation between intracranial metastasis and hippocampus was investigated to provide the theoretical foundation for the feasibility of hippocampal protection in the WBRT.

## Data and methods

### Data collection

#### Clinical data

The data of consecutive patients with brain metastasis admitted to our hospital from December 2014 to December 2016 were collected, including patients’ sex, age, and hospitalization number. Inclusion criteria: patients with brain metastasis; no radiotherapy contraindication; no history of radiation therapy in the brain. Exclusion criteria: patients with a history of cerebrovascular diseases affecting neurocognitive function within 3 months before radiotherapy; diagnosed mental illness and physical mental illness; congenital cognitive dysfunction. A total of 116 eligible patients were chosen, including 69 male patients and 47 female patients, with the median age of 64 years old (20–85 years old). Each patient had at least one intracranial metastatic lesion and at most 10 intracranial metastatic lesions.

#### Image data

The headrest and head thermoplastic mask model were used for head fixation. Computed tomography (CT) and Magnetic resonance imaging (MRI) scans were performed at the same position. Head CT and MRI images of the patients were transmitted to a Philips Pinnacle v9.8 treatment planning system in our department for image reconstruction and fusion processing at a later period. Images included axial view, sagittal view and coronal view.

#### Metastatic tumors and hippocampal sketch

**Fig. 1:**
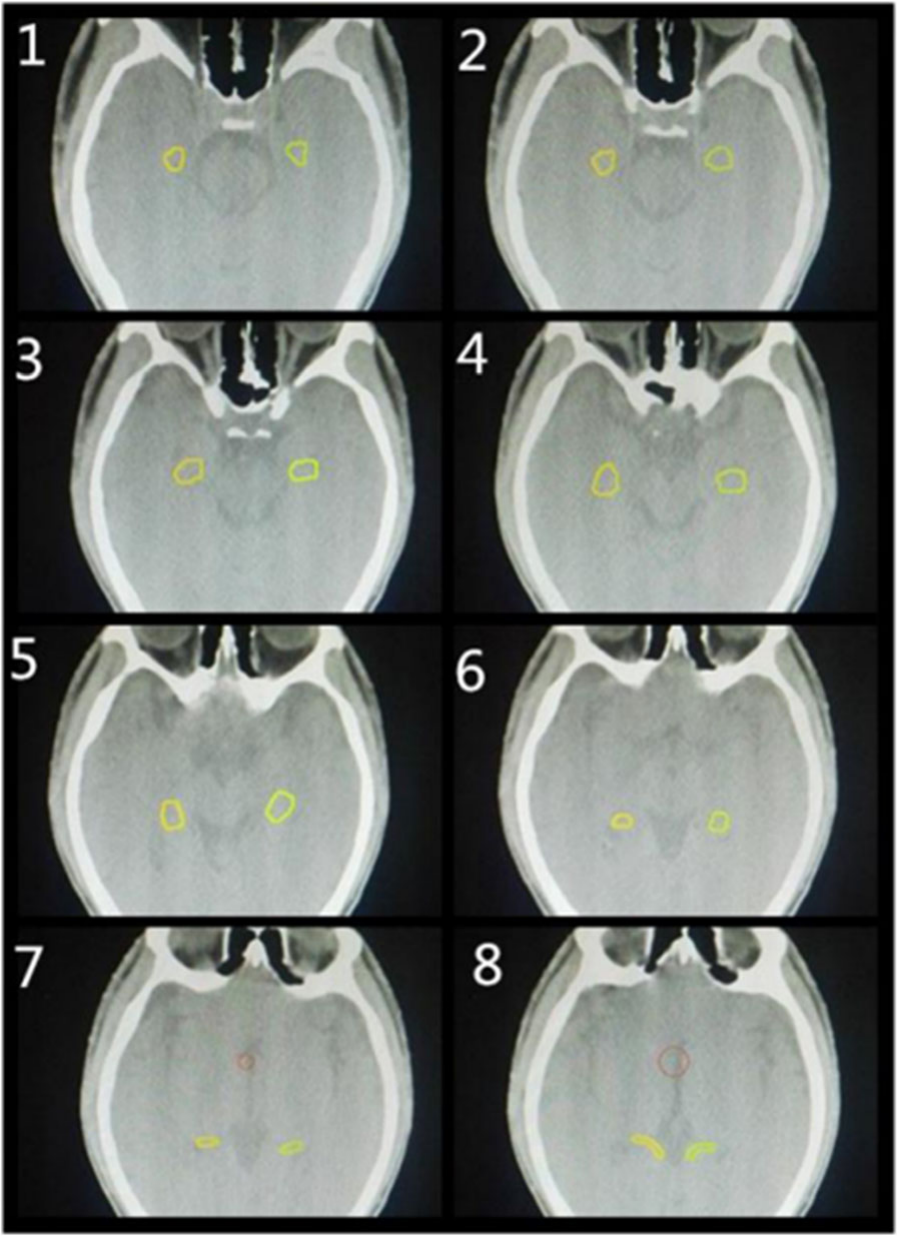
Axial images of the hippocampus contour based on the MRI-CT images fusion

All targets and organs at risk were delineated on the fusion images of simulation computed tomography and diagnostic MRI. Metastatic tumor gross volume was defined as the gross intracranial lesion on T1-weighted enhancing axial images and clinical target volume as whole brain. Hippocampal contouring was based on the RTOG 0933 research trial by the Radiation Therapy Oncology Group (RTOG) of the American College of Radiology (ACR). The hippocampus and metastatic tumors were contoured in the axial view. The contouring of the hippocampus was performed as follows: (1) the crescent bottom of the ventricular temporal angle was the start of the contour. The low-density grey matter was contoured on the low signals of the cerebrospinal fluid (CSF); (2) The grey matter (amygdaloid nucleus and hamulus) in the fimbria and the front of the tip temporal angle in the paracele was avoided; (3) The incidence of the temporal angle and amygdaloid nucleus fossa in the paracele defined the leading edge of the hippocampus; (4) The contouring of the cephalad was additionally performed, defining the inner side of the hippocampus as the lateral border of the corpora quadrigemina pond; (5) The hippocampal tail was still located in the back of the thalamus and bended to the callosum in the inner side; (6) The hippocampal tail was lengthened from the cephalad to the anteromedial paracele; (7) When the T1 low signal structure was not close to the paracele margin, the hippocampal contour was complete [20]. The lower parts of the hippocampus were contoured, while the T1 low signal grey matter area in the lower paracele was contoured. The contour of the hippocampal image of the axial horizon is shown in Fig. 1. After the bilateral hippocampus was contoured, the “region of interest expansion” was applied to automatically generate the 3D boundary of the 5-mm, 10-mm and 15-mm areas around the hippocampus. The sagittal view and coronal view images were automatically generated by a computer.

#### Research project

Each patient’s sex, age, primary tumors, Karnofsky performance status (KPS), total volume of metastatic lesions, brain’s average volume and the relation between the hippocampus and brain metastasis in its 5-mm surrounding area were investigated. We analyzed the factors having an impact on the hippocampus and brain metastasis in its 5-mm surrounding area for different tumor types and the internal relation between these factors. Also, the probability and overall distribution of metastatic tumors with different distances from the hippocampus were compared.

### Research methods

#### Operational methods

**Fig. 2:**
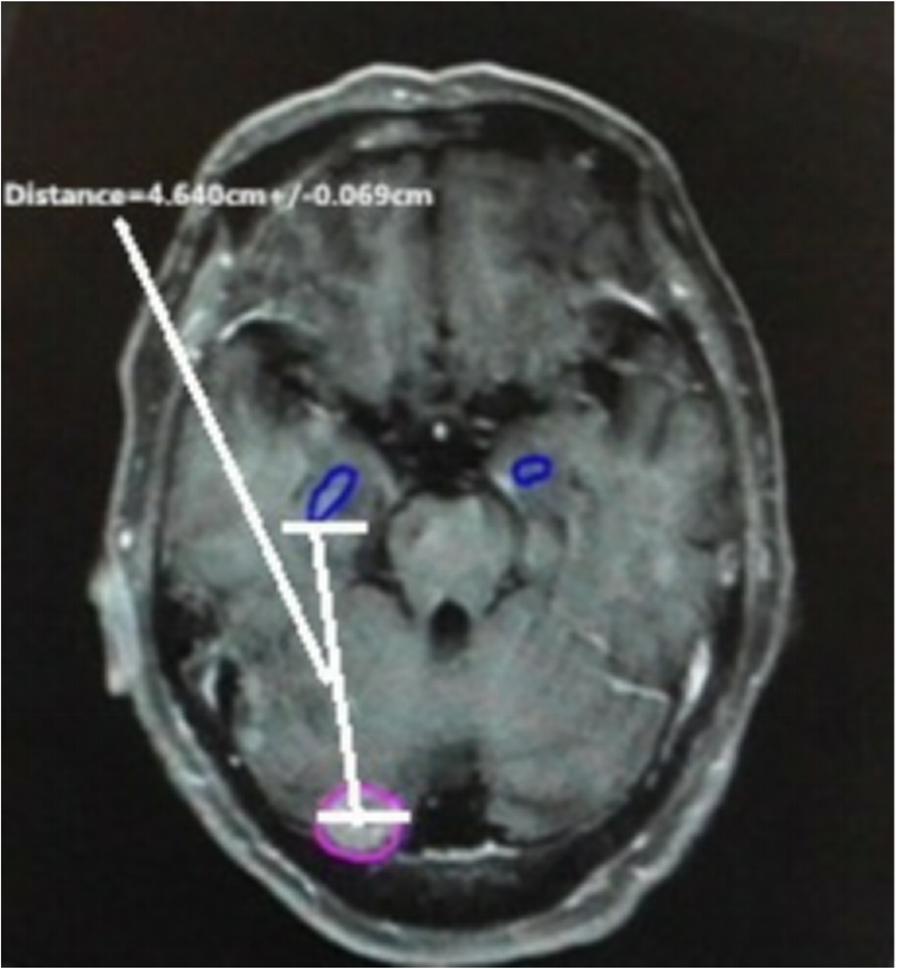
Minimum distance of intracranial metastases to the hippocampus

The clinical data of each patients were used to conduct the retrospective analysis. The number of intracranial metastatic tumors, metastatic volume, brain volume of each patient and the proportion of patients with different primary tumors were calculated to determine the minimal distance from each intracranial metastatic tumor center to the hippocampal boundary (According to RTOG 0933 research trial) (Fig. 2), taking the average. The number of intracranial metastatic tumors in the hippocampus and its 5-mm surrounding area was recorded. A binary variable logistic retrospective statistical method was used to analyze metastatic tumor parameters in the hippocampus and its 5-mm surrounding area and the relation between patients’ parameters.

#### Statistical analysis methods

The SPSS16.0 software was used to conduct the binary variable logistic regression analysis of the patients’ relevant data. Two-sided *P* < 0.05 showed the statistical difference.

## Results

A total of 565 metastatic lesions were contoured in the 116 eligible patients included in this study. The patients’ age, sex, KPS, primary tumors, total volume of metastatic lesions and brain volume were each recorded and are shown in Table 1. According to the cancer primary site the patients were distributed as follows: 59 with NSCLC, 19 with SCLC, 18 with breast cancer, 8 with rectal cancer and 12 with other tumors. Among these patients, there were 69 males and 47 females, the age of 53 patients was equal to or greater than 60 years old, and the KPS of 108 patients was equal to or greater than 70 scores. The median age of patients was 64 years old (20–85 years old). In addition, the number of total intracranial metastatic lesions was at least 1 and at most 10.

**Table 1:** Patients characteristics

Characteristics	Number(n)	Percentage(%)
Age (years)		
≥ 60	53	45.7
< 60	63	54.3
Gender		
Male	69	59.5
Female	41	40.5
KPS		
≥ 70	108	93.1
< 70	8	6.9
Primary site		
Non-small-cell lung cancer	59	50.9
Small-cell lung cancer	19	16.4
Breast	18	15.5
Colorectal	8	6.9
Other	12	10.3
Aggregate volume of intra-cranial \metastases (mm3)		
≥ 5500	64	55.2
< 5500	52	44.8
Aggregate volume of whole brain(m3)		
≥ 1.5	84	72.4
< 1.5	32	27.6

**Fig. 3:**
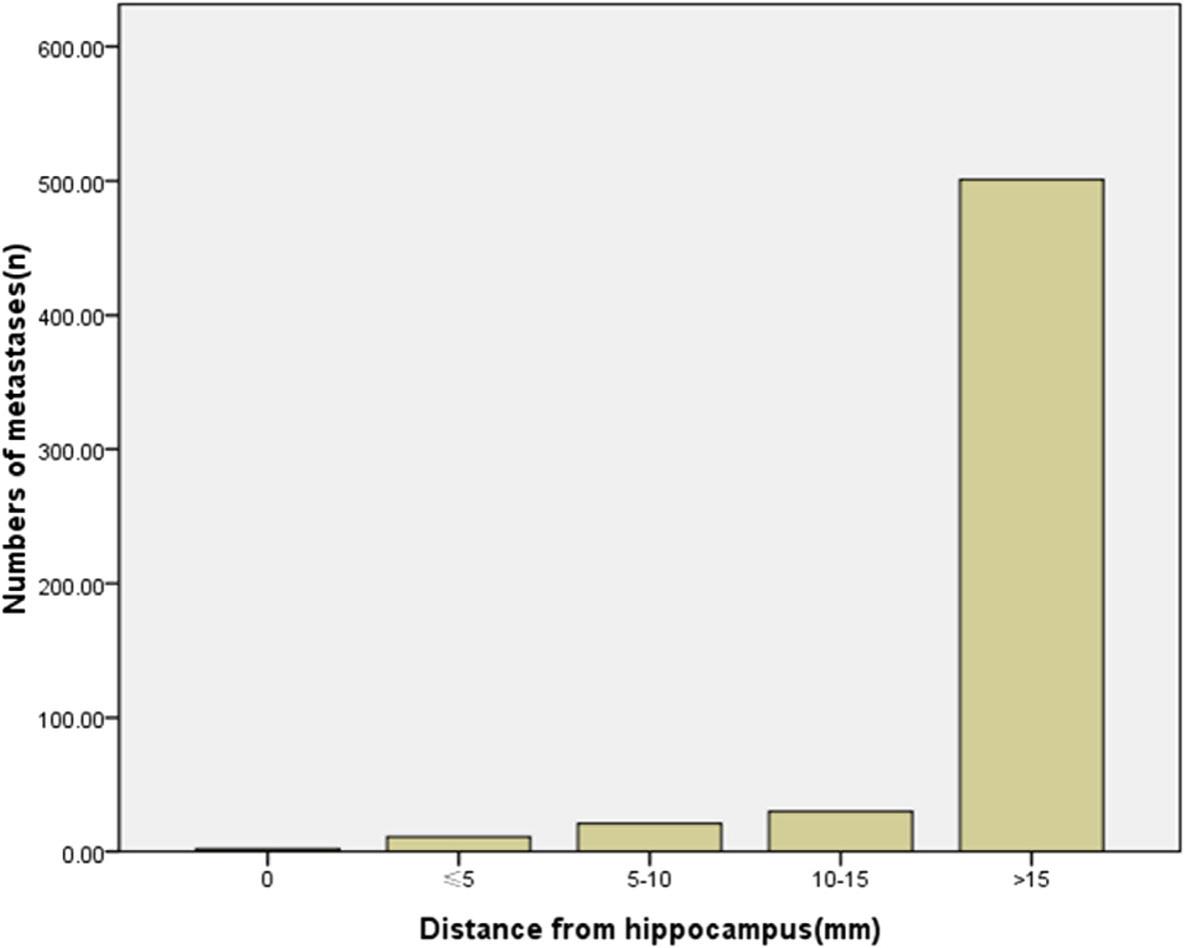
Distribution of metastatic disease in the brain in relation to the hippocampus

Among these 565 metastatic lesions, 2 patients had intracranial metastatic tumors in the hippocampus and primary tumors included NSCLC and SCLC. Two (0.4%) metastatic lesions in the hippocampus are shown in Fig. 3. There were 11 (1.9%) metastatic lesions in the area within 5 mm of the hippocampus, 21 (3.7%) metastatic lesions within 5–10 mm and 30 (5.3%) metastatic lesions within 10–15 mm. The remaining 501 (88.7%) metastatic lesions were located outside of the 15-mm surrounding area of the hippocampus. Overall, 11.2% (*n* = 13) of the patients had metastatic lesions in the hippocampus and its 5-mm surrounding area. Most of these patients were NSCLC patients (*n* = 5), while patients with rectal cancer and other tumors had the least metastatic lesions in the hippocampus. The distance distribution of intracranial metastatic tumors relative to the hippocampus is shown in Fig. 3, revealing that the distribution of intracranial metastatic lesions was uneven. Most of the metastatic tumors were present in areas outside the 15-mm area surrounding the hippocampus. The most frequent metastatic site was the frontal lobe (37.1%, *n* = 43), while 59.5% (*n* = 69) of the patients’ metastatic lesions were located outside the 15-mm area of the metastatic lesions, including 37 NSCLC patients.

Also, a binary logistic regression analysis method was used to analyze various parameters, including age (*P* = 0.395; dominance ratio = 0.467, 95% confidence interval (CI) = 0.081–2.704), sex (*P* = 0.139; dominance ratio = 0.259, 95% CI = 0.043–1.553), KPS (*P* = 0.724; dominance ratio = 1.502, 95% CI = 0.157–14.344), primary tumors (*P* = 0.894), total volume of metastatic lesions (*P* = 0.093; dominance ratio = 0.663, 95% CI = 0.410–1.072), and brain volume (*P* = 0.998; dominance ratio = 0.998, 95% CI = 0.989–1.007). The influences of each factor on the hippocampus and metastatic tumor occurrence within its 5-mm area were analyzed (Table 2). There was no statistical significance (*P* > 0.05, 95% CI).

**Table 2:** Binary logistic regression analysis of the incidence of metastases within the 5-mm area surrounding the hippocampus

Characteristics	OR	95%CI	P
Age	0.467	0.081–2.704	0.395
Sex	0.259	0.043–1.553	0.139
Primary site (NSCLC as reference)			0.894
SCLC	1.036	0.093–11.5	
Breast	0.708	0.047–10.77	
Colorectal	2.177	0.133–35.597	
Others	1.894	0.075–47.887	
KPS	1.502	0.157–14.344	0.724
Aggregate volume of intra-cranial metastases	0.663	0.410–1.072	0.093
Aggregate volume of whole brain	0.998	0.989–1.007	0.606

## Discussion

WBRT is a commonly used clinical treatment modality for intracranial primary tumors and intracranial metastatic tumors. Indeed, PCI has even become a standard treatment modality. European Organization for Research on Treatment of Cancer (EORTC) proves that relative to patients who receive WBRT at the very beginning, the progressive risks of metastatic lesions for patients without radiotherapy can be increased by 70–300% [9, 21]. Previously, due to the poor performance of radiotherapy in the treatment of intracranial metastatic tumors or intracranial primary tumors, the patients’ life expectancy was not long, thus damage to the central nervous system caused by the radiation receives insufficient concern, especially for forward damage. In fact, with the advance of radiotherapy and gradual extension of the patients’ life expectancy, the central lesions caused by radiation are gradually appeared, and they have gradually been noticed by patients, physicians and society. The hippocampus is a very important structure and it manages memory and cognition in humans. The hippocampal formation includes the Cornu Ammonis (CA1, CA2, CA3)and fascia dentate gyrus. Each area in the hippocampus is closely related with each other, but they develop a unique role in the information handling process of the learning memory [22]. Unfortunately, WBRT inevitably will cause hippocampal function damage. Neurocognitive impairment is likely the most common sequela of WBRT, especially for short-term memory dysfunction. Patients who have been exposed to WBRT for several months or even several years may have progressive impairment of the memory, spatial relation, visual motor process, quantity analysis as well as hypofunction in attention. Some scholars believe that the severity of the cognitive failure is determined by the exposure dose in the middle temporal lobe. The new memory formation in humans is related to the lifelong mitosis and neural stem cell areas—lower dentate gyrus being sensitive to radioactive rays. Clinical studies indicate that there is a dose-response relation between the hippocampal exposure dose and postradiotherapy recall hypomnesis. The studies confirmed that exceeding the 40% 7.3 Gy exposure dose of the bilateral hippocampal volume in fractionated exposure is related to long-term hypomnesis [2].

Intracranial metastatic tumors are the most common intracranial tumors in adults and the main causes for disease progression and death of terminal cancer patients. It is reported that about 10–20% of malignant tumor patients will ultimately develop into intracranial metastatic tumors of different primary lesions [11, 12]. Most patients with intracranial metastasis have a poor prognosis. About 50% of patients die of intracranial metastatic tumors, instead of out-cranial neoplasm [13]. The lung cancer morbidity is showing a downtrend in developed countries, but in developing countries it is rising and has tendency towards the younger population [23]. Lung cancer patients are usually in the later stage when they make a definite diagnosis, so the WBRT is of great importance in intracranial metastatic tumors, especially for intracranial metastasis of lung cancer. The WBRT can be conducted for the effective local control of intracranial lesions with the more operative difficulty and to alleviate local pressure symptoms, such as encephaledema, hemiplegia, etc. At the same time, the MRI of the head can be periodically performed in the radiotherapy process. According to the lesion progression situation, the treatment dose and boost in late stages should be timely adjusted.

Intensity-modulated radiation therapy (IMRT), tomotherapy (TOMO) and volumetric modulated arc therapy (VMAT) are widely applied in in the clinical treatment of patients nowadays. IMRT is the most extensively used technology and can take into account organ protection and dose requirements in the target region. Under the precondition of protecting normal organs and tissues, tumor cells can be killed to improve the patients’ quality of life and increase tumor control rate [20].TOMO has advantages in patients with an extensive range or multiple metastatic lesions and reduces hippocampal exposure dose by 87%, which is reduced to 0.49 Gy/F; IMRT can reduce hippocampal exposure dose by 81%, which is decreased to 0.72 Gy/F [20].

In this research, the spatial position of the intracranial metastatic tumors and the spatial relation with hippocampus were analyzed, taking into account two risks of hippocampal evasion: 1. The metastatic rate probability within the 5-mm area around the hippocampus was recorded. In clinical work, the external expansion of 5 mm in hippocampal evasion considers the possible errors and displacement in treatment; 2. Influences of the hippocampal contour and the boundary on the target region dose were rationally and accurately sketched. Gondi et al. selected 371 patients with intracranial metastasis to outlined the hippocampal areas for statistical analysis, and found that in a total of 1133 metastatic lesions in the 371 patients, the number of metastatic lesions within the 5-mm area around the hippocampus accounted for 3% of the total metastatic lesions and the number of patients with these lesions accounted for 8.6% of the total number of patients (95% CI = 5.7–11.5%). No metastatic lesion of the patients was present in the hippocampus [24]. After analyzing 856 metastatic lesions in 100 patients, Harth found that only 3% of patients had hippocampal metastatic lesions [16]. A retrospective analysis of 272 metastases in 100 patients by Ghia. Of the 272 identified metastases, 3.3% were within 5 mm of the hippocampus, and 86.4% of metastases were greater than 15 mm from the hippocampus [19]. In this study, 2 patients, whose primary tumor was a NSCLC in one and a SCLC in the other, had hippocampal metastasis. The occurrence of hippocampal metastatic tumors was 0.4%. In addition, 97.7% of intracranial metastatic lesions were located in the 5-mm area around the hippocampus. Among the primary tumors, the patients with lung cancer had the maximal proportion of intracranial metastatic lesion, followed by breast cancer and colorectal cancer. The above-mentioned proportion were consistent with the reported domestic and overseas research data. The occurrence of intracranial metastasis within the 5-mm area around the hippocampus was not related with patients’ age, sex, volume of metastatic lesions, brain volume, primary tumors and KPS.

In this research, the *P* value of the age for patients with metastasis within the 5-mm area around the hippocampus was 0.395 and the OR value was 0.467, indicating that the age was an irrelevant factor. Previously, studies on large samples indicated that there was a relation between the age and the 5-mm area around the hippocampus (patients with intracranial metastatic tumors below 60 years old were more prone to develop hippocampal metastasis) [25]. However, when a binary logistic regression analysis method similar to the one employed in our research was used to calculate patients’ metastatic lesions outside the 15-mm area around the hippocampus, the opposite conclusion was reached. Specifically that, patients with intracranial metastatic tumors above 60 years old had the larger probability of having metastatic lesions outside of the 15-mm area around the hippocampus, with *P* = 0.02 and OR = 3.663, which indicated to us that: patients with intracranial metastatic tumors below 60 years old had the larger probability of metastatic lesion in the 5-mm area around the hippocampus, while patients with intracranial metastatic tumors above 60 years old had the larger probability of metastatic lesions outside of the 15-mm areas around the hippocampus. Currently, there is no definite agreement about the influence of age on metastatic tumors with regard to different distances in the hippocampus, predicting that age may be closely related to the prognosis of patients with intracranial metastasis. Relevant studies show that age is an adverse factor for the prognosis of patients with intracranial metastasis [26, 27]. The younger patients have the higher probability of progressive disease, due to their longer life expectancy, thus the potential possibility of hippocampal metastatic lesion or recurrence is higher. Moreover, studies show that with the increase of age, adult patients suffer more severe damage caused by radiotherapy [28]. In addition, due to the elder overall age of the patients, other diseases can be added to improve different treatment methods for patients with intracranial metastatic tumors in different ages, so as to treat patients with a purpose and optimize the treatment purpose.

Among the 116 patients with intracranial metastasis in our research, there were 47 females, which included 18 patients with breast cancer. Additionally, 3 patients had intracranial metastatic tumors within the 5-mm area around the hippocampus. In addition, the total number of patients’ metastatic lesions was greater than or equal to 4. It is widely known that breast cancer is a common cancer in women, which shows a yearly increasing morbidity. Moreover, the age of onset of this cancer shows a tendency towards younger women. Epidemiological research shows that breast cancer is the second most common tumor with brain metastasis behind the lung cancer [29]. Whole brain exposure is the leading treatment modality for patients with breast cancer who have at least 4 intracranial metastatic lesions, but it cannot improve the overall survival of patients [30]. Other studies show that breast cancer patients with at least 10 intracranial metastatic lesions have the higher probability of intracranial metastatic tumors within the 5-mm area around the hippocampus [31]. When patients with oligonucleotide metastatic lesions (1–3 metastatic lesions) received WBRT avoiding the hippocampus, the metastatic risk in the hippocampus was lower than that in patients with non-oligonucleotide metastatic lesions [18], indicating that the number of total intracranial metastatic lesions might be related to hippocampal metastasis. The higher the number of intracranial metastatic lesions is, the higher probability of hippocampal metastasis in primary breast cancer patients will be. It was determined that among all patients with intracranial metastasis, NSCLC patients with intracranial metastasis had the largest proportion (50.9%, *n* = 59), which was greater than that of the other primary tumors. Data from countries other than China, show that NSCLC in later stage is a common pathogenesis in the central nervous system and about 24–44% of them with the progressive disease will develop into intracranial metastasis [32]. Due to the presence of the blood brain barrier (BBB), the systematic therapeutic effects of intracranial metastatic tumors are not ideal, thus excision, WBRT and gamma knife surgery become primary treatment modalities for intracranial metastatic tumors intervention. Clinically, numerous NSCLC patients with intracranial metastasis select the WBRT and they often suffer neurocognitive function damage after treatment by the intracranial radiotherapy, resulting in reduction of their quality of life. In the clinical treatment process, NSCLC patients should appreciate hippocampal protection.

Preclinical and clinical evidence suggests that radiation dose received by the neural stem cells of the subgranular zone in the hippocampus may play a role in radiation-induced neurocognitive decline, specifically memory recall. There is a differential sensitivity to WBRT of various memory-related neurocognitive domains. This provides the rationale to explore the clinical feasibility of hippocampal sparing during WBRT [33]. Also, children’s neurocognitive function damage is related to the neural stem cells and temporal lobe exposure dose. The younger infant patients will suffer from more severe damage caused by the radiotherapy. The exposure range of child patients < 3 years old with medulloblastoma receiving radiotherapy is clearly less than that of child patients > 3 years old. Even so, there is still relatively severe cognitive damage [34]. Studies show that women’s untoward effects generated by head radiotherapy are more severe than those in men, but the mechanism is not clear. In order to study such a phenomenon, Roughton et al [35] used C57BL/6J mice of different sex to conduct an experiment and hypothesized that: (1) For men and women who received head radiotherapy, the lower dentate gyrus had damage and also had certain irreversibility; (2) After radiotherapy, the phenomenon of cell proliferation reduction in the granule cell layer was more obvious in females; (3) After men and women were treated with the radiotherapy, the spatial moving ability was slightly enhanced, while the research ability was weakened, and accompanied with symptoms of anxiety. The above-mentioned phenomena were more obvious in women; (4) After women were treated with radiotherapy, the spatial station-keeping ability was reduced, but such a phenomenon did not occur in men, indicating that after inspecting radiotherapy effects in clinical work, sex should be considered, thus patients with secondary radiotherapy should pay more attention to hippocampal protection.

This study drew the following conclusion: the possibility of metastatic tumors within the 5-mm area surrounding the hippocampus might be unrelated with patients’ age, sex, KPS, primary tumors, volume of metastatic lesions and brain volume. The probability of hippocampal metastatic rate was very low. Only 11.2% of patients had 1 (11 patients) or 2 (2 patients) brain metastatic lesions located in the 5-mm area around the hippocampus, indicating that it might be feasible to implement hippocampal avoidance during head radiotherapy. It is predicted that 88.8% of patients will benefit from WBRT avoiding the hippocampus. Due to sample size deviation, the proportion of patients with colorectal cancer who had hippocampal metastatic lesions was lower than that with lung cancer patients, thus a conclusion could not be drawn about whether WBRT avoiding the hippocampus could be implemented in lung cancer patients. However, this study still had imperfections and limitations: (1) The hippocampal contouring overseas stipulates that the thickness of the positioning image layer should be less than or equal to 1.25 mm. Accordingly, to provide convenience for the integration between the MRI image and CT image, the positioning image thickness was 3 mm. Furthermore, the thinner the image layer thickness is, the more accurate the hippocampal contouring will be, thus the hippocampal contouring in this study was not sufficiently accurate. (2) The sample size in this study was small, thus significant data deviation might exist. The credibility interval of each research factor was larger; thus the accuracy might have certain influence on the study results; (3). With territoriality in data acquisition, the metastatic morbidity of local lung cancer was much higher than that of other diseases. (4). The research design was a single-central small sample study, thus the comparative analysis of population data with different stages is required to improve the accuracy of the conclusions.

## Conclusions

This study revealed that for patients with intracranial metastatic tumors, the probability of developing metastasis within the 5-mm area around the hippocampus was low. Additionally, there was no obvious correlation between the probability of intracranial metastasis within the 5-mm area around the hippocampus and each of the factors evaluated. Hippocampal-avoidance WBRT may be applied to patients with brain metastases.

## Abbreviations

ACR: American College of Radiology
BBB: Blood brain barrier
BM: Brain metastases
CA: Cornu Ammonis
CSF: Cerebrospinal fluid
CT: Computed tomography
HA: Hippocampal avoidance
IMRT: Intensity-modulated radiation therapy
KPS: Karnofsky performance
MRI: Magnetic Resonance imaging
NSCLC: Non-small cell lung cancer
PCI: Prophylactic cranial irradiation
PHM: Perihippocampal metastases
RTOG: Radiation Therapy Oncology Group
SCLC: Small cell lung cancer
TOMO: Tomotherapy
VMAT: Volumetric modulated arc therapy
WBRT: Whole-brain radiotherapy
